# Are TREK Channels Temperature Sensors?

**DOI:** 10.3389/fncel.2021.744702

**Published:** 2021-10-06

**Authors:** Lola Rueda-Ruzafa, Salvador Herrera-Pérez, Ana Campos-Ríos, J. A. Lamas

**Affiliations:** ^1^CINBIO, Laboratory of Neuroscience, University of Vigo, Vigo, Spain; ^2^Laboratory of Neuroscience, Galicia Sur Health Research Institute (IISGS), Vigo, Spain; ^3^Grupo de Neurofisiología Experimental y Circuitos Neuronales, Hospital Nacional de Parapléjicos, SESCAM, Toledo, Spain

**Keywords:** TREK channels, temperature sensors, potassium channels, DRG, nodose ganglion

## Abstract

Internal human body normal temperature fluctuates between 36.5 and 37.5°C and it is generally measured in the oral cavity. Interestingly, most electrophysiological studies on the functioning of ion channels and their role in neuronal behavior are carried out at room temperature, which usually oscillates between 22 and 24°C, even when thermosensitive channels are studied. We very often forget that if the core of the body reached that temperature, the probability of death from cardiorespiratory arrest would be extremely high. Does this mean that we are studying ion channels in dying neurons? Thousands of electrophysiological experiments carried out at these low temperatures suggest that most neurons tolerate this aggression quite well, at least for the duration of the experiments. This also seems to happen with ion channels, although studies at different temperatures indicate large changes in both, neuron and channel behavior. It is known that many chemical, physical and therefore physiological processes, depend to a great extent on body temperature. Temperature clearly affects the kinetics of numerous events such as chemical reactions or conformational changes in proteins but, what if these proteins constitute ion channels and these channels are specifically designed to detect changes in temperature? In this review, we discuss the importance of the potassium channels of the TREK subfamily, belonging to the recently discovered family of two-pore domain channels, in the transduction of thermal sensitivity in different cell types.

## Introduction

The last large family of potassium channels discovered, two-pore domain potassium channels (K2P), is composed of 6 subfamilies of which the so-called TREK (TWIK-1 related K channels) is modulated by a large number of physiological stimuli, including temperature. Importantly the broad expression of this subfamily includes the sensory components of the autonomic, peripheral and central nervous systems, in particular the nodose, trigeminal and dorsal root ganglia, besides other places related with temperature homeostasis such as the hypothalamus (Fink et al., 1996, 1998; Maingret et al., 2000; Meadows et al., 2000, 2001; Hervieu et al., 2001; Matsumoto et al., 2001; Medhurst et al., 2001; Talley et al., 2001; Gu et al., 2002; Alloui et al., 2006; Kang and Kim, 2006; Kang et al., 2006; Yamamoto et al., 2009; Cadaveira-Mosquera et al., 2012; Lamas, 2012; Pereira et al., 2014; Conti, 2018; Fernández-Fernández et al., 2018; Viatchenko-Karpinski et al., 2018). A pending issue in this regard is determining the expression of TREK channels in the terminals (nerve endings) of thermoreceptor neurons where the first stage of sensory transduction takes place. Traditionally, the cells that transduce the different stimuli are called sensory receptors, so that the cells that capture changes in temperature are called temperature receptors or thermoreceptors. With the discovery of the molecular mechanism for uptake of stimuli, the first molecule that detects the stimulus is often called a receptor, so the term thermoreceptor has become confusing. Along this review, the ion channels that detect the thermal stimulus are called sensors or detectors, that is, thermosensors or thermodetectors and the cells expressing them thermoreceptors.

In mammals, control of the central region temperature is essential for survival. Drastic variations in thermal homeostasis range have been associated with feverish, inflammatory, hypothermic and hyperthermic processes (Bouchama et al., 1991; Planas et al., 1995). Pyrogens entrance into the blood in a febrile process generates an immune response carried mainly by macrophages, which produce proinflammatory cytokines that stimulate the production of prostaglandin E2 (PGE2) in hypothalamus, inducing cAMP release, which in turn raises the level of the hypothalamic thermostat to around 39°C (Dinarello, 1999). Although the peripheral temperature control is less life threatening, the most studied system is that of capturing the skin temperature through the dorsal root ganglion afferents (mainly A-delta and C fibers). This system, although less critical, is essential because it provides information on the ambient temperature rather than on the body itself. In recent years, the interest in the mechanism of thermal information uptake by the autonomic nervous system has increased, especially by the sensory neurons of the nodose ganglion. In this case the information, although for the most part unconscious, comes from internal organs such as the viscera and the central nervous system and should be critical to keep internal temperature constant.

Both, the broad distribution of TREK channels and their great sensitivity to temperature changes make these channels clear candidates to be good temperature sensors, similar to what happens with some cationic transient receptor potential channels (TRPs). In this article, we will review the molecular and cellular mechanisms underlying this important function of TREK channels.

## Q10 and Temperature Ranges

The first member of the TREK subfamily discovered was TREK1, and in the seminal article by Fink et al. (1996) the biophysical properties of this channel were studied in injected oocytes and transfected COS cells at room temperature. The same approach was used when TRAAK (Fink et al., 1998) and TREK2 (Bouchama et al., 1991) were revealed. However, after their discovery, all three members (TREK1, TREK2, and TRAAK) were demonstrated to be strongly sensitive to temperature. The first mention of TREK temperature sensitivity was introduced for TREK1 by Maingret and coauthors (Maingret et al., 2000).

Now it is generally accepted that the activity of all three TREK channels is essentially absent at temperatures below ∼10°C, very low at room temperature (22–24°C), increases gradually when increasing bath temperature, is strong (about half-maximum) at physiological temperatures (∼30°C), maximum at about 40 °C and then decreases again (Maingret et al., 2000; Kang et al., 2005; Kang and Kim, 2006; Zhang et al., 2008; Noël et al., 2009; Schneider et al., 2014; Woo et al., 2019). Importantly, the effect of increasing temperature is not voltage-dependent, but heat-activated TREK1 currents (37°C) show a strong outwardly rectifying current/voltage (IV) relationship reversing at the equilibrium potential for potassium and the same is true for TREK2 and TRAAK (Maingret et al., 2000; Kang et al., 2005; Bagriantsev et al., 2012; Woo et al., 2016).

We should keep in mind that the temperature range that affects individual ion channels does not always match with the temperature range at which these channels affect animal behavior. When we talk about animals, we usually talk about four thermal sensations to which four temperature ranges have been assigned. Although the temperature ranges may vary from one author to another, the following are representative: cold (−10 to 15°C), cool (16 to 30°C), warm (31 to 42°C) and hot (43 to 60°C) (Lamas et al., 2019). Cold and hot temperatures may be noxious or painful and hence aversive. Interestingly, leak currents observed in dorsal root ganglion (DRG) neurons at room- (22 °C) and warm-temperatures (30°C) can be suppressed by TREK-channel blockers like riluzole and norfluoxetine, at 14°C the effect of these blockers was negligible as TREK channels were already closed by cold temperatures (Viatchenko-Karpinski et al., 2018). Puzzling, temperature activation (37°C) produced a strong reduction in norfluoxetine sensitivity of heterologously expressed TREK2 currents, indicating that the effect of fluoxetine may be very different at 37°C compared with room temperature (McClenaghan et al., 2016).

The activity of ion channels is often modified by changes in temperature, but how sensitive does a channel need to be in order to be considered a thermosensor? There is general agreement that there is a special dependence on temperature when Q10 exceeds a value of about 3 (warm sensitive) or is well below 1 (cool sensitive) (Brauchi et al., 2004; Wechselberger et al., 2006; Vriens et al., 2014; Wang and Siemens, 2015; Conti, 2018; Buijs and McNaughton, 2020). When dealing with TREK1 channels, a temperature increment of 10°C enhances TREK1 currents by ∼7–9 fold, being more sensitive in the range 32–37°C and similar or even higher (∼20 fold) sensitivities to temperature were reported for TREK2 and TRAAK (Maingret et al., 2000; Kang et al., 2005). It is important to say that all these characteristics can be observed in heterologously expressed (COS, HEK, oocytes) or native (cerebellar neurons, DRG neurons, cardiac myocytes, astrocytes) TREK channels and in most recording configurations, saving excised patches (see section “Molecular Mechanism”) (Maingret et al., 2000; Kang et al., 2005; Kang and Kim, 2006; Kreneisz et al., 2009; Kucheryavykh et al., 2009; Bagriantsev et al., 2012). It is worth noting that other K2P channels, like TASK or THIK, have not shown a comparable temperature dependency (Rajan et al., 2001; Kang et al., 2005; Bagriantsev et al., 2011).

## Warm and Hot

### C-Fibers

Using an isolated skin-saphenous nerve preparation, Alloui et al. showed that slowly conducting C-fibers, from control mice, increased the firing of action potentials when the temperature was increased (20 s heating ramps from 30 to 50°C). Additionally, C-fibers from TREK1 knockout (KO) mice fired more action potentials, in response to heat ramps, than their wild counterparts (Alloui et al., 2006). The same was confirmed in TREK2-KO, triple-KO (TREK1/TREK2/TRAAK-KO) (Pereira et al., 2014), TRAAK-KO and TREK1/TRAAK-KO mice (Noël et al., 2009). Interestingly, in TREK2-KO mice the firing of C-fibers is affected only below 40°C (warm temperatures) but C-fibers from triple-KO mice still keep the firing enhancement at hot temperatures (40–50°C), indicating that TREK2 must deal with warm but not with hot temperatures. For a summary of these data and those that follow, see Table 1.

**TABLE 1:** Summary of the effects of knocking out TREK channels in c-fibers, DRG neurons and freely moving animals when changing temperature.

	TREK1 *KO*	TREK2 *KO*	TRAAK *KO*	TREK1/TRAAK *KO*	TREK2/TRAAK *KO*	TREK1/TREK2/TRAAK *KO*	References
C-fibers: n° AP, Temp.: 30–50°C	↑	↑	↑	↑		↑	Alloui et al., 2006; Pereira et al., 2014
C-fibers: n° AP, Temp.: 40–50°C		→				↑	Pereira et al., 2014
C-fibers: Firing Threshold Temp.: 30–50 °C	↓	↓	↓			↓	Alloui et al., 2006; Pereira et al., 2014; Noël et al., 2009
% C-fibers responding to warming Temp.: 30–50°C	↑	↑	↑	↑		↑	Alloui et al., 2006; Pereira et al., 2014; Noël et al., 2009
% DRG responding to heat Temp.: 50 °C	↑		↑	↑			Noël et al., 2009
Tail withdrawal. Latency Temp.: 40–50 °C	↓		↓	↓			Alloui et al., 2006; Pereira et al., 2014
Tail withdrawal. Latency Temp.: 44–46°C		↓				↓	Noël et al., 2009
Hot Plate. Latency Temp.: 50–52 52–56°C	→	↓	→			↓	Alloui et al., 2006; Pereira et al., 2014; Noël et al., 2009
Tail-flick reflex. Latency Temp.: 40–42°C		↓				↓	Pereira et al., 2014
% DRG responding to cold Temp.: 10°C	→		→	↑			Noël et al., 2009
C-fibers n° AP, Temp.: 31–>10°C	→	↑		↑		↑	Alloui et al., 2006; Pereira et al., 2014; Noël et al., 2009
% C-fibers responding to cold Temp.: 31 to 10°C	→	↑	→	↑		↑	Descoeur et al., 2011; Pereira et al., 2014
Tail withdrawal. Latency Temp.: 20–25°C	→	↓	↑	↓		↓	Alloui et al., 2006; Pereira et al., 2014; Noël et al., 2009; Descoeur et al., 2011
Tail withdrawal. Latency Temp.: 5–15°C		→				↓	Pereira et al., 2014
Cold plate. Latency	→		→	↑			Alloui et al., 2006; Pereira et al., 2014; Noël et al., 2009; Descoeur et al., 2011
Temp Perefernce test. Time at 30°C 25–30°C						↑	Noël et al., 2009; Descoeur et al., 2011
Oxaliplatin neuropathic model. Cold hypersensitivity				↑	→		Pereira et al., 2014

*↑: increase, ↓: decrease, →: no effect.*

*C-fibers: n° AP, Temp.: 31- >.*

The threshold for heat induced spike firing was also lower in TREK1-KO mice (36 against 42°C), suggesting that TREK1 may be involved in the transduction of noxious heat, similar results were obtained in TRAAK-KO and double KO mice (TREK1/TRAAK-KO) (Alloui et al., 2006; Noël et al., 2009). The threshold was also reduced for TREK2- and triple-KO (∼35°C) mice when compared with wild mice (∼40°C) (Pereira et al., 2014).

Also the percentage of C-fibers responding to warming (30 to 50°C) was increased in TREK1-KO, TRAAK-KO, TRAAK/TREK1-KO mice (Alloui et al., 2006; Noël et al., 2009), TREK2-KO and triple-KO showing an increased proportion of heat-sensitive (50°C) C-fibers (Pereira et al., 2014).

Although not all combinations of KO mice have been tested (Table 1), existing data indicate that a reduction in TREK channels has at least three effects on type C afferent fibers. Firstly, the percentage of afferents that respond to warming/heating increases. Secondly, the firing threshold using a heating ramp decreases. Finally, the number of action potentials triggered when the afferents are heated increases. All these compared to the heat response of C-fibers from wild mice.

It has recently been shown that the Ranvier nodes of the Aβ-afferents of the trigeminal nerve express TREK1 and TRAAK channels, but not TREK2 (Kanda et al., 2019). The leakage current through these channels and their open probability clearly increases when passing from 24 to 35°C. On the contrary, reducing the temperature from 24 to 10°C reduces the activity of the TREK channels and the amplitude of the leakage current. It would be interesting to know if the TREK channels of the nodes influence in any way the capture of thermal information. At the moment we know that the reduction in temperature (35 to 15) progressively reduces the conduction velocity of trigeminal afferents, probably due to the reduction in the activity of the TREK channels of the node of Ranvier.

### DRG Neurons

The cell bodies of the C fibers are located in the dorsal root and trigeminal ganglia and, accordingly, the percentage of cultured DRG neurons responding (calcium influx) to noxious heat (30 to 50°C) strongly increased from 34% in wild type (WT) to 64% in TREK1-KO, 60% in TRAAK-KO and 74% in double KO mice (Noël et al., 2009). Interestingly, the TREK2 activator T2A3 reduced the increase in calcium normally observed in DRG neurons in response to aversive temperatures (40°C) (Dadi et al., 2017). As expected, these data corroborated the data obtained in C-fibers with similar temperature ranges.

It was also reported that TREK channels contributed about 85% of the potassium background current present in DRG neurons at 37°C, interestingly the major contributors were TREK2 channels (69%) followed by TREK1 (12%) and TRAAK (3%) (Kang and Kim, 2006). Consistently, TREK2 knockdown (siRNA C) has been shown to depolarize small rat DRG neurons by about 10 mV, suggesting a major role of TREK2 channels on the resting membrane potential (RMP) at 37 °C (Acosta et al., 2014). However, at 24°C, TREK channels showed a very low activity and other K2P channels (TRESK) became more relevant at the resting level (Kang and Kim, 2006; Rivas-Ramírez et al., 2020a). The reason why different authors disagree on which of the two channels, TREK1 or TREK2 is more abundant in DRG neurons is unknown. It has been suggested that the difference in species and age of the animals used or the presence/absence of nerve growth factor (NGF) in the culture medium could account for this difference (Alloui et al., 2006). It is possible that the different techniques used (electrophysiological, neuroanatomical and/or molecular biology techniques) are also involved in this controversy.

### Nodose Ganglion

Deep thermoreceptors of the viscera collect thermal information through sensory neurons with somas located in the nodose ganglion (NG) and this information ascend to the hypothalamus via the nucleus of the solitary tract (Morrison et al., 2008; Romanovsky et al., 2009). To our knowledge, the relationship among NG neurons, TREK channels and temperature has not been investigated, however, the presence of TREK channels in most NG neurons has been reported (Zhao et al., 2010; Cadaveira-Mosquera et al., 2012; Fernández-Fernández et al., 2018).

### Central Structures

As we have said above, the hypothalamus is a central structure that plays an essential role in the regulation of information related to temperature. The hypothalamus contains a good number of thermosensitive neurons dedicated to detect the temperature of the hypothalamus itself (Abe et al., 2003; Tabarean et al., 2005; Conti, 2018). Most of these thermosensitive neurons fired spontaneously and were considered warm sensitive, a low percentage were cold sensitive. In a first approach the expression of TREK1 in the hypothalamus was reported to be very low (Fink et al., 1996), but the same group reported later a good expression of this channel in regions of the hypothalamus implicated in thermoregulation like preoptic and anterior hypothalamus. TRAAK channels have also been reported to have a low expression in the hypothalamus (Fink et al., 1998; Wechselberger et al., 2006). Although there may be some discrepancy in relation to the degree of expression or the neuronal type, there is general agreement in the presence of the three members of the TREK family in the hypothalamus (Lamas, 2012).

### Behavioral Tests

TREK1-KO mice are hypersensitive to thermal pain between 46 and 50°C (short tail withdrawal latency) (Alloui et al., 2006). Although the range of temperatures is no completely clear, both TRAAK-KO and TRAAK/TREK1-KO mice showed heat hyperalgesia (Noël et al., 2009). It should be taken in mind that TREK channels are active at normal skin temperatures and contribute to the RMP of sensory neurons. Interestingly, TREK2-KO seems to affect the withdrawal latency only below 46°C (44–46°C), suggesting again that TREK2 may be important in the warm range of temperatures while TREK1 and TRAAK would be more implicated at hot temperatures (Pereira et al., 2014).

TREK2-KO and triple-KO showed a similar enhancement of sensibility to warm unaversive temperatures (∼40–42°C, tail flick reflex) (Pereira et al., 2014). It is not clear why TREK1-KO or TRAAK-KO mice do not show increased sensibility in the hot-plate test like the TREK1/TRAAK-KO does (Alloui et al., 2006; Noël et al., 2009), but compounds enhancing the activity of TREK1 channels have been shown to display analgesic activity using the same test (Vivier et al., 2017). Also TREK2-KO behaved like WT mice in the hot-plate test at 50–52°C while the triple KO mice showed a clear thermal hypersensitivity (Pereira et al., 2014).

**FIGURE 1:**
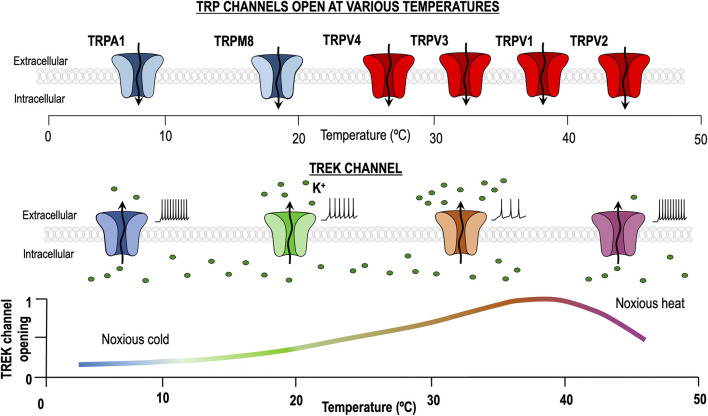
Dual role of TREK channels in thermosensation. At physiological temperature (37°C), TREK channels are maximally activated, allowing K+ to pass through them and generating a hyperpolarizing leak current. Temperature decrease and noxious heat processes reduce the TREK channels open probability, hence increasing excitability. By contrast, transient receptor potential (TRP) channels activated by heating and cooling are different entities and their activity is minimal around 30°C.

In summary TREK channels seem to be more important for heat/pain transduction in the range of ∼35 to 45°C (Alloui et al., 2006; Honore, 2007). It has been proposed that the heat-induced hyperpolarization produced by the activation of TREK channels may serve as a counterbalance for the heat-induced depolarization resulting from the activation of TRPV1 channels, and hence playing an important role in heat sensing (Figure 1) (Schneider et al., 2014). Accordingly, all three TREK channels have been shown to co-localize strongly with thermosensitive TRP channels in trigeminal and dorsal root ganglion cells (Nealen et al., 2003; Yamamoto et al., 2009; La et al., 2011).

## Cool and Cold

### C-Fibers

C-fibers from TREK1-KO responded (increasing action potential firing) much like those from wild mice when a cold stimulus was applied to the skin (31 to 10°C) (Alloui et al., 2006). However, it has been strongly suggested that TREK channels could be good cold sensors (Maingret et al., 2000; Kang et al., 2005; Wechselberger et al., 2006). Supporting this suggestion, double KO, but not the single TREK1- or TRAAK-KO mice, showed an increased percentage of C-fibers responding to cold stimuli (30 to 10°C) and the firing of action potentials of these C-fibers was increased when compared with those obtained from wild type mice (Noël et al., 2009). Similarly, both TREK2-KO and triple-KO mice showed an increase in the percentage of C-fibers responding to cold and in their firing rate (Pereira et al., 2014).

### DRG Neurons

When cooling cultured rat DRG neurons from 32 to 20°C a depolarization of about 10 mV, occasionally accompanied of action potential firing, occurs in 25 % of them (Reid and Flonta, 2001). It has been reported that this depolarization is due to the inhibition of a background potassium current called I_cold_, and that this current could be transported through TREK1 channels (Reid and Flonta, 2001). Consistently, Maingret et al. reported intense TREK1 staining in small and medium sized DRG neurons (Maingret et al., 2000) and later, a good expression of all three members of the TREK subfamily was shown in small sized DRG neurons (Viatchenko-Karpinski et al., 2018). Similarly, a small percentage (∼10 %) of cultured mouse trigeminal ganglion neurons are activated (increase of intracellular calcium, depolarization and action potential firing) by lowering temperature, this sensitivity was present in small diameter neurons (Viana et al., 2002). Voltage clamp experiments demonstrated that the increase in excitability provoked by cooling involved the closure of a resting potassium current, probably transported by TREK1 channels (Viana et al., 2002). Accordingly, TREK1 channels seems to be better expressed than TREK2 and TRAAK in DRG neurons (Alloui et al., 2006).

It is accepted that about 25% of small diameter DRG neurons are activated by cold (12°C, calcium influx) (Noël et al., 2009), this percentage falls to 10% of the neurons of the mouse DRG in culture (Vetter et al., 2013). Interestingly, sympathetic neurons of the superior cervical ganglion and many DRG/TG neurons can be intrinsically activated by cold stimuli through a mechanism not depending on TRP (TRPM8, TRPA1) channels (Thut et al., 2003; Smith et al., 2004; Munns et al., 2007; Buijs and McNaughton, 2020). Similarly, c-Fos activity is increased in Grueneberg ganglion neurons when mice pups are exposed to 22°C for 2 h and this effect is weaker in TREK1-KO mice but independent of TRPs (Stebe et al., 2014). Although the basis of this behavior is not totally understood, the presence of TREK channels may contribute to it (Reid and Flonta, 2001; Viana et al., 2002; Munns et al., 2007; Cadaveira-Mosquera et al., 2011, 2012; Vetter et al., 2013; MacDonald et al., 2020; Rivas-Ramírez et al., 2015, 2020a,b). Consistently, mice lacking both TREK1 and TRAAK channels (but not those lacking only one of them) showed a percentage of small diameter DRG neurons responding to cold higher (54%) that their wild counterparts (Noël et al., 2009). On the contrary, experiments carried out in rat TG neurons suggested that TREK1 may not have a prominent role in cold transduction (Thut et al., 2003).

### Nodose Ganglion

A large fraction of rat and mouse nodose neurons are activated (Intracellular calcium increase, depolarization and action potential firing) by cold stimuli (Fajardo et al., 2008). Although the cationic TRPA1 channels seem to play a major role in this activation, the expression of TREK channels at this level could be relevant and deserves to be investigated.

### Central Structures

The three members of the TREK subfamily are expressed in the hippocampus (Talley et al., 2001; Lamas, 2012) and cooling evoked network activation in this structure has been ascribed to the presence of TREK channels, mainly to the closure of TREK1 channels (de la Pena et al., 2012).

### Behavioral Tests

When lowering temperature and looking at behavioral tests, the latency in the tail withdrawal test and the response in the cold plate assay were comparable between single TREK1- or TRAAK-KO and WT mice (Alloui et al., 2006; Noël et al., 2009). On the contrary, the double KO (TREK1/TRAAK-KO) showed increased sensitivity to cold in the cold plate and thermal-preference assay (Noël et al., 2009; Descoeur et al., 2011). Also, TREK2-KO and triple-KO mice showed an increased sensitivity (lower latency) to temperatures of 20–25°C, being stronger in triple-KO (Pereira et al., 2014). Interestingly, when using noxious cold temperatures (5–15°C) in a tail immersion test, only the triple-KO but not the TREK2-KO showed lower latencies than wild-type mice (Pereira et al., 2014), suggesting that TREK2 may be important in the cool range of temperatures, but not for the sensitivity to noxious cold. Similar to what happens with heat sensitivity, the TREK1 and TRAAK channels seem to be related to the uptake of stimuli in the cold range (aversive), whereas the TREK2 would regulate the uptake of stimuli in the cool temperature range (harmless).

### Neuropathic Cold

In a neuropathic model, it has been shown that double KO (TREK1/TRAAK-KO) mice showed a high cold hypersensitivity when compared with WT or TRAAK-KO mice (Noël et al., 2009). In the same line, it has been proposed that cold hypersensitivity and allodynia induced by oxaliplatin, a cancer chemotherapy (Beijers et al., 2015), is due at least in part to the reduction in the expression of TREK1 and TRAAK channels (Descoeur et al., 2011; Poupon et al., 2018). Indeed, allodynia provoked by oxaliplatin is very similar to that observed in TREK1/TRAAK-KO mice and oxaliplatin cannot further enhance the allodynia produced by the double KO (Descoeur et al., 2011). Interestingly, oxaliplatin did not modified the temperature (30–20°C) preference of TREK2-KO and triple-KO mice (Pereira et al., 2014). When dealing with colder temperatures (15–17°C, cold plate), oxaliplatin exacerbated the perception of cold in TREK2-KO but not in triple-KO mice (Pereira et al., 2014).

Riluzole, an activator of TREK channels (Duprat et al., 2000; Lesage et al., 2000; Cadaveira-Mosquera et al., 2011; Fernández-Fernández et al., 2018; Lamas and Fernandez-Fernandez, 2019), reduced the nociceptive response to cold temperatures in WT, TREK2-KO and TRAAK-KO but not in TREK1 and triple-KO mice (Poupon et al., 2018). Riluzole has also been shown to prevent the oxaliplatin-induced cold-hypersensitivity by acting on TREK1 channels, as this analgesic effect was lost in TREK1-KO mice and in animals treated with spadin, a TREK1 inhibitor (Poupon et al., 2018). Notwithstanding, it should be taken in mind that both the sensitivity to cold and the oxaliplatin hypersensitivity are lost in TRPM8-KO mice and that the expression of other thermosensitive channels may also be altered by oxaliplatin (Descoeur et al., 2011).

## Molecular Mechanism

Temperature increase in the range of 24 to 42°C do not affect the single channel conductance of TREK channels, but it clearly increases the open probability and reduces the open-time duration (Kang et al., 2005). The C-terminal region of TREK1 seems to be the main temperature-sensing element in TREK1 channels because partial deletion of this terminal (Δ103) or substitution of the C-terminal region for that of TASK1 strongly reduces the activation of the channel by heat (Maingret et al., 2000; Bagriantsev et al., 2011, 2012). On the contrary deletion of the N-terminal zone did not affect temperature stimulation (Maingret et al., 2000). Interestingly, replacement of TREK2 C-terminal with that of TASK3 did not reduced its thermosensitivity (Kang et al., 2005) but deletion of the C-terminal domain strongly reduced its temperature sensitivity (McClenaghan et al., 2016).

Besides the important role of the C-terminal domain for TREK1 heat sensitivity, it has been proposed that the C-type gate mediates temperature sensitivity of TREK1 and TREK2. This hypothesis, deeply reviewed by Schneider, suggests that the intracellular C-terminal domain works as the sensor capturing the information of the thermal stimulus and transfers it to the extracellular C-type gate through the M4 transmembrane segment and that this mechanism could be shared by other (i.e., mechanical) stimuli (Bagriantsev et al., 2011, 2012; Schneider et al., 2014). These studies have also suggested that mutations in the M4 segment reduced the response of TREK1 channels to temperature by uncoupling the sensor (intracellular C-terminal region) from the gating apparatus (extracellular C-type gate) (Bagriantsev et al., 2011).

The C-terminal end of TREK is also required for its sensitivity to various pharmacological agonists and physical stimuli (Maingret et al., 1999). Although information about TREK channel behavior at temperatures close to physiological (32–37°C) is very scarce, we know that at 37°C all three members of the subfamily respond to arachidonic acid, pH changes and membrane stretch, when they are recorded in cell-attached (with the electrode attached to the membrane) or inside out (exposing the cytosolic side to the bath medium) patches, much like they do at room temperature (Kang et al., 2005; Kang and Kim, 2006). To our knowledge, most of the inhibitors and activators of TREK channels work in a similar way at room temperature and at 30°C (Cohen et al., 2009; Kreneisz et al., 2009). TREK1 channels are activated by membrane stretch, this activation is greatly amplified when the temperature is increased (about 10-fold by 20°C), interestingly, such an effect is lost when the channels are recorded in excised patches (Maingret et al., 2000). Actually, it has been suggested that temperature and stretch may act synergistically and share the same mechanism to increase the activity of TREK channels, acting on the C-terminal domain and hence regulating the C-type gate (Schneider et al., 2014). On the other hand, the enhancement of TREK1 currents by temperature is strongly reduced by an increase in external osmolarity (Maingret et al., 2000). Native TREK2 currents in cortical astrocytes have been shown to increase with temperature. This increment was much bigger in astrocytes subjected to simulated ischemia (Kucheryavykh et al., 2009). These and other data strongly indicate a complex interaction among different modulators of TREK channel activity, interaction that deserves to be investigated more deeply.

Surprisingly, all TREK channels fail to be activated by increasing temperature when they are recorded in excised outside-out (the cytosolic side of the cell is in contact with the pipette solution) or inside-out configurations. This indicates that thermal modulation of TREK1, TREK2 and TRAAK needs the cell to be intact and suggests the participation of an internal “second messenger” (Maingret et al., 2000; Kang et al., 2005; Honore, 2007). Accordingly, all TREK1, TREK2 and TRAAK keep their thermosensitivity (in the range of 24 to 42°C) when recorded in cell-attached patches (Maingret et al., 2000; Kang et al., 2005). Whether the channels themselves are temperature sensitive remains to be unequivocally demonstrated.

Interestingly, the activation of protein kinase A (PGE-cAMP-PKA) and resulting phosphorylation of Ser333 reduces the enhancement of TREK1 currents when increasing temperature (Maingret et al., 2000). On the contrary, intracellular ATP did not affect the increase of heterologously expressed TREK2 currents provoked by changing temperature from 26 to 37°C (Woo et al., 2016).

## Concluding Remarks and Perspectives

Due to the discovery of the molecular mechanisms that take place in the thermal stimuli reception, the traditional term thermoreceptor has become a confusing concept to refer to the neurons responsible for the thermal transduction. As we have clarified in the introduction, a thermoreceptor is a sensory receptor that transduces the specific stimuli caused by temperature changes, and the first molecule that detects the thermal stimulus is called thermosensor or thermodetector.

Another type of confusion can be caused by using the terms “heat receptor” and “cold receptor.” Generally, a neuron is called a heat receptor if it increases its excitability (firing frequency) when increasing the temperature, if this happens when the temperature is lowered, we would call it a cold receptor. Can we use the same reasoning to talk about sensors? If so, a cold sensor should increase the activity (open probability) when lowering temperature. Paradoxically, the TREK channels increase their activity (opening probability) with increasing temperature, but they are often called cold sensors (Maingret et al., 2000). This is explained because they are potassium channels open at physiological temperatures and that they hyperpolarize the thermoreceptor when activated. A cold thermoreceptor should increase its excitability when the temperature decreases and TREK channels reduce their activity, hence transporting cold information (Buijs and McNaughton, 2020). In summary a heat sensor activates a cold receptor.

To understand how TREK and cold sensitive TRP channels can cooperate to sense cold stimuli let’s assume that at physiological temperature, about 30 °C, the activity of TREK channels is reasonably high (Figure 1), while the activity of cold sensitive TRPs (and TRPs in general) is very low (Noël et al., 2009). In this situation both channel types, if coexpressed, would tend to drive the cold receptor far from the threshold and hence we would expect the receptor to be hyperpolarized and silent. When the temperature is reduced (from 30 to 10°C), TREK channel activity would fall to zero and the activity of cold sensitive TRPs would strongly increase. At this point, both channels tend to depolarize the cold thermoreceptor which in turn should increase firing and send the information to the next step. It seems clear that both TREK1 and TRAAK are important to regulate the excitability of DRG thermosensitive neurons as removal of both is necessary to observe a clear effect in the process of cold sensing.

Table 1 summarizes what happen in TREK KO models, but what should we expect to happen in a complete TREK-KO mice? In the receptors from KO mice, at 30°C the lack of TREK channels would cease to exert a hyperpolarizing effect, which would be maintained exclusively by the lack of activity of the TRPs and other background channels. It would then be expected that at 30°C the thermoreceptor would slightly increase its activity. At 10°C, however, the situation in the two models (WT and TREK-KO) should be similar since in both cases the TREK channels are out of play and the TRP channels are activated. This hypothesis could explain the higher number of action potentials observed in the C fibers of KO mice when cooling, since they would have a higher basal firing before cooling.

Apart from their role as thermosensors in thermoreceptor neurons, TREK channels, due to their wide expression, contribute to the maintenance of the RMP and to the control of excitability in a good number of cell types not related to general thermoreception. Chick embryonic atrial myocytes show a rather positive RMP (around −20 mV) at room temperature, increasing the temperature to 35°C hyperpolarized these cells to −70 mV due to the activation of TREK-like (TREK1 and TREK2) potassium channels (Zhang et al., 2008). Surprisingly, cultured DRG neurons from TREK1-KO mice were reported to show the same RMPs than the neurons obtained from WT animals, unfortunately this experiment was carried out at room temperature and not repeated at more physiological temperatures (Alloui et al., 2006). It is worth noting that the contribution of TREK channels to the RMP maintenance cannot be fully appreciated at room temperature since they have practically no activity in these conditions (Rivas-Ramírez et al., 2020a).

We have more and more evidence of the importance of TREK channels as thermosensors, however, most of the studies carried out to investigate other important roles of these channels have been carried out at room temperature. We should make an effort to reinvestigate the importance of these channels at temperatures closer to what these channels are subjected to in a living organism. A central and still open question is whether TREK channels are just regulating neuronal excitability or they really represent a cold-transduction mechanism.

## Author Contributions

JL: conceptualization, validation, resources, writing—review and editing, supervision, and funding acquisition. AC-R, LR-R, SH-P, and JL: investigation. JL, LR-R, and SH-P: writing—original draft preparation. All authors have read and agreed to the published version of the manuscript.

## Conflict of Interest

The authors declare that the research was conducted in the absence of any commercial or financial relationships that could be construed as a potential conflict of interest.

## Publisher’s Note

All claims expressed in this article are solely those of the authors and do not necessarily represent those of their affiliated organizations, or those of the publisher, the editors and the reviewers. Any product that may be evaluated in this article, or claim that may be made by its manufacturer, is not guaranteed or endorsed by the publisher.

## References

[B1] Abe J. Okazawa M. Adachi R. Matsumura K. Kobayashi S. (2003). Primary cold-sensitive neurons in acutely dissociated cells of rat hypothalamus. *Neurosci. Lett.* 342 29–32. 10.1016/S0304-3940(03)00239-8 12727310

[B2] Acosta C. Djouhri L. Watkins R. Berry C. Bromage K. Lawson S. N. (2014). TREK2 expressed selectively in IB4-binding C-fiber nociceptors hyperpolarizes their membrane potentials and limits spontaneous pain. *J. Neurosci.* 34 1494–1509. 10.1523/JNEUROSCI.4528-13.2014 24453337 PMC3898302

[B3] Alloui A. Zimmermann K. Mamet J. Duprat F. Noel J. Chemin J. (2006). TREK-1, a K+ channel involved in polymodal pain perception. *EMBO J.* 25 2368–2376. 10.1038/sj.emboj.7601116 16675954 PMC1478167

[B4] Bagriantsev S. N. Clark K. A. Minor D. L. Jr. (2012). Metabolic and thermal stimuli control K(2P)2.1 (TREK-1) through modular sensory and gating domains. *EMBO J.* 31 3297–3308. 10.1038/emboj.2012.171 22728824 PMC3411076

[B5] Bagriantsev S. N. Peyronnet R. Clark K. A. Honore E. Minor D. L. Jr. (2011). Multiple modalities converge on a common gate to control K2P channel function. *EMBO J.* 30 3594–3606. 10.1038/emboj.2011.230 21765396 PMC3181481

[B6] Beijers A. J. Mols F. Tjan-Heijnen V. C. Faber C. G. van de Poll-Franse L. V. Vreugdenhil G. (2015). Peripheral neuropathy in colorectal cancer survivors: the influence of oxaliplatin administration. Results from the population-based PROFILES registry. *Acta Oncol.* 54 463–469. 10.3109/0284186X.2014.980912 25417732

[B7] Bouchama A. Parhar R. S. el-Yazigi A. Sheth K. al-Sedairy S. (1991). Endotoxemia and release of tumor necrosis factor and interleukin 1 alpha in acute heatstroke. *J. Appl. Physiol.* 70 2640–2644. 10.1152/jappl.1991.70.6.2640 1885459

[B8] Brauchi S. Orio P. Latorre R. (2004). Clues to understanding cold sensation: thermodynamics and electrophysiological analysis of the cold receptor TRPM8. *Proc. Natl. Acad. Sci. U.S.A.* 101 15494–15499. 10.1073/pnas.0406773101 15492228 PMC523461

[B9] Buijs T. J. McNaughton P. A. (2020). The role of cold-sensitive ion channels in peripheral thermosensation. *Front. Cell Neurosci.* 14:262. 10.3389/fncel.2020.00262 32973456 PMC7468449

[B10] Cadaveira-Mosquera A. Perez M. Reboreda A. Rivas-Ramirez P. Fernandez-Fernandez D. Lamas J. A. (2012). Expression of K2P channels in sensory and motor neurons of the autonomic nervous system. *J. Mol. Neurosci.* 48 86–96. 10.1007/s12031-012-9780-y 22544515

[B11] Cadaveira-Mosquera A. Ribeiro S. J. Reboreda A. Perez M. Lamas J. A. (2011). Activation of TREK currents by the neuroprotective agent riluzole in mouse sympathetic neurons. *J. Neurosci.* 31 1375–1385. 10.1523/JNEUROSCI.2791-10.2011 21273422 PMC6623616

[B12] Cohen A. Sagron R. Somech E. Segal-Hayoun Y. Zilberberg N. (2009). Pain-associated signals, acidosis and lysophosphatidic acid, modulate the neuronal K(2P)2.1 channel. *Mol. Cell Neurosci.* 40 382–389. 10.1016/j.mcn.2008.12.004 19130888

[B13] Conti B. (2018). Molecular basis of central thermosensation. *Handb. Clin. Neurol.* 156 129–133. 10.1016/B978-0-444-63912-7.00008-4 30454586

[B14] Dadi P. K. Vierra N. C. Days E. Dickerson M. T. Vinson P. N. Weaver C. D. (2017). Selective small molecule activators of TREK-2 channels stimulate dorsal root ganglion c-fiber nociceptor two-pore-domain potassium channel currents and limit calcium influx. *ACS Chem. Neurosci.* 8 558–568. 10.1021/acschemneuro.6b00301 27805811 PMC5901755

[B15] de la Pena E. Malkia A. Vara H. Caires R. Ballesta J. J. Belmonte C. (2012). The influence of cold temperature on cellular excitability of hippocampal networks. *PLoS One* 7:e52475. 10.1371/journal.pone.0052475 23300680 PMC3534091

[B16] Descoeur J. Pereira V. Pizzoccaro A. Francois A. Ling B. Maffre V. (2011). Oxaliplatin-induced cold hypersensitivity is due to remodelling of ion channel expression in nociceptors. *EMBO Mol. Med.* 3 266–278. 10.1002/emmm.201100134 21438154 PMC3377073

[B17] Dinarello C. A. (1999). Cytokines as endogenous pyrogens. *J. Infect. Dis.* 179 S294–S304. 10.1086/513856 10081499

[B18] Duprat F. Lesage F. Patel A. J. Fink M. Romey G. Lazdunski M. (2000). The neuroprotective agent riluzole activates the two P domain K+ channels TREK-1 and TRAAK. *Mol. Pharmacol.* 57 906–912. 10779373

[B19] Fajardo O. Meseguer V. Belmonte C. Viana F. (2008). TRPA1 channels mediate cold temperature sensing in mammalian vagal sensory neurons: pharmacological and genetic evidence. *J. Neurosci.* 28 7863–7875. 10.1523/JNEUROSCI.1696-08.2008 18667618 PMC6670374

[B20] Fernández-Fernández D. Cadaveira-Mosquera A. Rueda-Ruzafa L. Herrera-Pérez S. Veale E. L. Reboreda A. (2018). Activation of TREK currents by riluzole in three subgroups of cultured mouse nodose ganglion neurons. *PLoS One* 13:e0199282. 10.1371/journal.pone.0199282 29928032 PMC6013220

[B21] Fernández-Fernández D. Cadaveira-Mosquera A. Rueda-Ruzafa L. Herrera-Pérez S. Veale E. L. Reboreda A. (2018). Activation of TREK currents by riluzole in three subgroups of cultured mouse nodose ganglion neurons. *PLoS One* 13:e0199282. 10.1371/journal.pone.0199282 PMC6013220 29928032

[B22] Fink M. Duprat F. Lesage F. Reyes R. Romey G. Heurteaux C. (1996). Cloning, functional expression and brain localization of a novel unconventional outward rectifier K+ channel. *EMBO J.* 15 6854–6862. 10.1002/j.1460-2075.1996.tb01077.x 9003761 PMC452511

[B23] Fink M. Lesage F. Duprat F. Heurteaux C. Reyes R. Fosset M. (1998). A neuronal two P domain K+ channel stimulated by arachidonic acid and polyunsaturated fatty acids. *EMBO J.* 17 3297–3308. 10.1093/emboj/17.12.3297 9628867 PMC1170668

[B24] Gu W. Günter S. Jochen R. H. Hartmut E. Chiristine K. S. Andreas K. S. (2002). Expression pattern and functional characteristics of two novel splice variants of the two-pore-domain potassium channel TREK-2. *J. Physiol. Lond* 539 657–668. 10.1113/jphysiol.2001.013432 11897838 PMC2290188

[B25] Hervieu G. J. Cluderay J. E. Gray C. W. Green P. J. Ranson J. L. Randall A. D. (2001). Distribution and expression of TREK-1, a two-pore-domain potassium channel, in the adult rat CNS. *Neuroscience* 103 899–919. 10.1016/S0306-4522(01)00030-6 11301200

[B26] Honore E. (2007). The neuronal background K2P channels: focus on TREK1. *Nat. Rev. Neurosci.* 8 251–261. 10.1038/nrn2117 17375039

[B27] Kanda H. Ling J. Tonomura S. Noguchi K. Matalon S. Gu J. G. (2019). TREK-1 and TRAAK Are Principal K(+) Channels at the Nodes of Ranvier for Rapid Action Potential Conduction on Mammalian Myelinated Afferent Nerves. *Neuron* 104 960–971.e7. 10.1016/j.neuron.2019.08.042 31630908 PMC6895425

[B28] Kang D. Kim D. (2006). TREK-2 (K2P10.1) and TRESK (K2P18.1) are major background K+ channels in dorsal root ganglion neurons. *Am. J. Physiol. Cell. Physiol.* 291 C138–C146. 10.1152/ajpcell.00629.2005 16495368

[B29] Kang D. Choe C. Kim D. (2005). Thermosensitivity of the two-pore domain K+ channels TREK-2 and TRAAK. *J. Physiol. London* 564 103–116. 10.1113/jphysiol.2004.081059 15677687 PMC1456046

[B30] Kang D. Han J. Kim D. (2006). Mechanism of inhibition of TREK-2 (K2P10.1) by the Gq-coupled M3 muscarinic receptor. *Am. J. Physiol. Cell. Physiol.* 291 C649–C656. 10.1152/ajpcell.00047.2006 16672694

[B31] Kreneisz O. Benoit J. P. Bayliss D. A. Mulkey D. K. (2009). AMP-activated protein kinase inhibits TREK channels. *J. Physiol. London* 587 5819–5830. 10.1113/jphysiol.2009.180372 19840997 PMC2808542

[B32] Kucheryavykh L. Y. Kucheryavykh Y. V. Inyushin M. Shuba Y. M. Sanabria P. Cubano L. A. (2009). Ischemia increases TREK-2 channel expression in astrocytes: relevance to glutamate clearance. *Open Neurosci. J.* 2 40–47. 10.2174/1874082000903010040 19890471 PMC2771865

[B33] La J. H. Schwartz E. S. Gebhart G. F. (2011). Differences in the expression of transient receptor potential channel V1, transient receptor potential channel A1 and mechanosensitive two pore-domain K+ channels between the lumbar splanchnic and pelvic nerve innervations of mouse urinary bladder and colon. *Neuroscience* 186 179–187. 10.1016/j.neuroscience.2011.04.049 21549810 PMC3118582

[B34] Lamas J. A. (2012). “Mechanosensitive K2P channels, TREKking through the autonomic nervous system,” in *Mechanically Gated Channels and Their Regulation*, eds Kamkin A. Lozinsky I. (Dordrecht: Springer Science+Business Media), 35–68. 10.1007/978-94-007-5073-9_2

[B35] Lamas J. A. Fernandez-Fernandez D. (2019). Tandem pore TWIK-related potassium channels and neuroprotection. *Neural Regen Res.* 14 1293–1308. 10.4103/1673-5374.253506 30964046 PMC6524494

[B36] Lamas J. A. Rueda-Ruzafa L. Herrera-Perez S. (2019). Ion channels and thermosensitivity: TRP, TREK, or both? *Int. J. Mol. Sci.* 20:2371. 10.3390/ijms20102371 31091651 PMC6566417

[B37] Lesage F. Terrenoire C. Romey G. Lazdunski M. (2000). Human TREK2, a 2P domain mechano-sensitive K+ channel with multiple regulations by polyunsaturated fatty acids, lysophospholipids, and Gs, Gi, and Gq protein-coupled receptors. *J. Biol. Chem.* 275 28398–28405. 10.1074/jbc.M002822200 10880510

[B38] MacDonald D. I. Wood J. N. Emery E. C. (2020). Molecular mechanisms of cold pain. *Neurobiol. Pain* 7:100044. 10.1016/j.ynpai.2020.100044 32090187 PMC7025288

[B39] Maingret F. Lauritzen I. Patel A. J. Heurteaux C. Reyes R. Lesage F. (2000). TREK-1 is a heat-activated background K+ channel. *EMBO J.* 19 2483–2491. 10.1093/emboj/19.11.2483 10835347 PMC212769

[B40] Maingret F. Patel A. J. Lesage F. Lazdunski M. Honore E. (1999). Mechano- or acid stimulation, two interactive modes of activation of the TREK-1 potassium channel. *J. Biol. Chem.* 274 26691–26696. 10.1074/jbc.274.38.26691 10480871

[B41] Matsumoto I. Emori Y. Ninomiya Y. Abe K. (2001). A comparative study of three cranial sensory ganglia projecting into the oral cavity: in situ hybridization analyses of neurotrophin receptors and thermosensitive cation channels. *Brain Res. Mol. Brain Res.* 93 105–112. 10.1016/S0169-328X(01)00129-2 11589988

[B42] McClenaghan C. Schewe M. Aryal P. Carpenter E. P. Baukrowitz T. Tucker S. J. (2016). Polymodal activation of the TREK-2 K2P channel produces structurally distinct open states. *J. Gener. Physiol.* 147 497–505. 10.1085/jgp.201611601 27241700 PMC4886281

[B43] Meadows H. J. Benham C. D. Cairns W. Gloger I. Jennings C. Medhurst A. D. (2000). Cloning, localisation and functional expression of the human orthologue of the TREK-1 potassium channel. *Pflugers Arch.* 439 714–722. 10.1007/s004249900235 10784345

[B44] Meadows H. J. Chapman C. G. Duckworth D. M. Kelsell R. E. Murdock P. R. Nasir S. (2001). The neuroprotective agent sipatrigine (BW619C89) potently inhibits the human tandem pore-domain K(+) channels TREK-1 and TRAAK. *Brain Res.* 892 94–101. 10.1016/S0006-8993(00)03239-X 11172753

[B45] Medhurst A. D. Rennie G. Chapman C. G. Meadows H. Duckworth M. D. Kelsell R. E. (2001). Distribution analysis of human two pore domain potassium channels in tissues of the central nervous system and periphery. *Brain Res. Mol. Brain Res.* 86 101–114. 10.1016/S0169-328X(00)00263-1 11165377

[B46] Morrison S. F. Nakamura K. Madden C. J. (2008). Central control of thermogenesis in mammals. *Exp. Physiol.* 93 773–797. 10.1113/expphysiol.2007.041848 18469069 PMC2496891

[B47] Munns C. AlQatari M. Koltzenburg M. (2007). Many cold sensitive peripheral neurons of the mouse do not express TRPM8 or TRPA1. *Cell. Calcium.* 41 331–342. 10.1016/j.ceca.2006.07.008 16949152

[B48] Nealen M. L. Gold M. S. Thut P. D. Caterina M. J. (2003). TRPM8 mRNA is expressed in a subset of cold-responsive trigeminal neurons from rat. *J. Neurophysiol.* 90 515–520. 10.1152/jn.00843.2002 12634279

[B49] Noël J. Zimmermann K. Busserolles J. Deval E. Alloui A. Diochot S. (2009). The mechano-activated K+ channels TRAAK and TREK-1 control both warm and cold perception. *EMBO J.* 28 1308–1318. 10.1038/emboj.2009.57 19279663 PMC2683043

[B50] Pereira V. Busserolles J. Christin M. Devilliers M. Poupon L. Legha W. (2014). Role of the TREK2 potassium channel in cold and warm thermosensation and in pain perception. *Pain* 155 2534–2544. 10.1016/j.pain.2014.09.013 25239074

[B51] Planas M. E. Rodriguez L. Sanchez S. Pol O. Puig M. M. (1995). Pharmacological evidence for the involvement of the endogenous opioid system in the response to local inflammation in the rat paw. *Pain* 60 67–71. 10.1016/0304-3959(94)00090-2 7715943

[B52] Poupon L. Lamoine S. Pereira V. Barriere D. A. Lolignier S. Giraudet F. (2018). Targeting the TREK-1 potassium channel via riluzole to eliminate the neuropathic and depressive-like effects of oxaliplatin. *Neuropharmacology* 140 43–61. 10.1016/j.neuropharm.2018.07.026 30056126

[B53] Rajan S. Wischmeyer E. Karschin C. Preisig-Muller R. Grzeschik K. H. Daut J. (2001). THIK-1 and THIK-2, a novel subfamily of tandem pore domain K+ channels. *J. Biol. Chem.* 276 7302–7311. 10.1074/jbc.M008985200 11060316

[B54] Reid G. Flonta M. L. (2001). Cold transduction by inhibition of a background potassium conductance in rat primary sensory neurones. *Neurosci. Lett.* 297 171–174. 10.1016/S0304-3940(00)01694-3 11137755

[B55] Rivas-Ramírez P. Cadaveira-Mosquera A. Lamas J. A. Reboreda A. (2015). Muscarinic modulation of TREK currents in mouse sympathetic superior cervical ganglion neurons. *Eur. J. Neurosci.* 42 1797–1807. 10.1111/ejn.12930 25899939

[B56] Rivas-Ramírez P. Reboreda A. Rueda-Ruzafa L. Herrera-Pérez S. Lamas J. A. (2020a). Contribution of KCNQ and TREK channels to the resting membrane potential in sympathetic neurons at physiological temperature. *Int. J. Mol. Sci.* 21:5796. 10.3390/ijms21165796 32806753 PMC7461115

[B57] Rivas-Ramírez P. Reboreda A. Rueda-Ruzafa L. Herrera-Pérez S. Lamas J. A. (2020b). PIP2 mediated inhibition of TREK potassium currents by bradykinin in mouse sympathetic neurons. *Int. J. Mol. Sci.* 21:389. 10.3390/ijms21020389 31936257 PMC7014146

[B58] Romanovsky A. A. Almeida M. C. Garami A. Steiner A. A. Norman M. H. Morrison S. F. (2009). The transient receptor potential vanilloid-1 channel in thermoregulation: a thermosensor it is not. *Pharmacol. Rev.* 61 228–261. 10.1124/pr.109.001263 19749171 PMC2763780

[B59] Schneider E. R. Anderson E. O. Gracheva E. O. Bagriantsev S. N. (2014). Temperature sensitivity of two-pore (K2P) potassium channels. *Curr. Top. Membr.* 74 113–133. 10.1016/B978-0-12-800181-3.00005-1 25366235 PMC4794111

[B60] Smith M. P. Beacham D. Ensor E. Koltzenburg M. (2004). Cold-sensitive, menthol-insensitive neurons in the murine sympathetic nervous system. *Neuroreport* 15 1399–1403. 10.1097/01.wnr.0000126559.35631.54 15194861

[B61] Stebe S. Schellig K. Lesage F. Breer H. Fleischer J. (2014). The thermosensitive potassium channel TREK-1 contributes to coolness-evoked responses of *Grueneberg ganglion* neurons. *Cell Mol. Neurobiol.* 34 113–122. 10.1007/s10571-013-9992-x 24101433 PMC11488964

[B62] Tabarean I. V. Conti B. Behrens M. Korn H. Bartfai T. (2005). Electrophysiological properties and thermosensitivity of mouse preoptic and anterior hypothalamic neurons in culture. *Neuroscience* 135 433–449. 10.1016/j.neuroscience.2005.06.053 16112471

[B63] Talley E. M. Solorzano G. Lei Q. Kim D. Bayliss D. A. (2001). CNS distribution of members of the two-pore-domain (KCNK) potassium channel family. *J. Neurosci.* 21 7491–7505. 10.1523/JNEUROSCI.21-19-07491.2001 11567039 PMC6762917

[B64] Thut P. D. Wrigley D. Gold M. S. (2003). Cold transduction in rat trigeminal ganglia neurons *in vitro*. *Neuroscience* 119 1071–1083. 10.1016/S0306-4522(03)00225-2 12831865

[B65] Vetter I. Hein A. Sattler S. Hessler S. Touska F. Bressan E. (2013). Amplified cold transduction in native nociceptors by M-channel inhibition. *J. Neurosci.* 33 16627–16641. 10.1523/JNEUROSCI.1473-13.2013 24133266 PMC6618521

[B66] Viana F. de la Peña E. Belmonte C. (2002). Specificity of cold thermotransduction is determined by differential ionic channel expression. *Nat. Neurosci.* 5 254–260. 10.1038/nn809 11836533

[B67] Viatchenko-Karpinski V. Ling J. Gu J. G. (2018). Characterization of temperature-sensitive leak K(+) currents and expression of TRAAK, TREK-1, and TREK2 channels in dorsal root ganglion neurons of rats. *Mol. Brain* 11:40. 10.1186/s13041-018-0384-5 29980241 PMC6035395

[B68] Vivier D. Soussia I. B. Rodrigues N. Lolignier S. Devilliers M. Chatelain F. C. (2017). Development of the first two-pore domain potassium channel TWIK-related K(+) channel 1-selective agonist possessing *in vivo* antinociceptive activity. *J. Med. Chem.* 60 1076–1088. 10.1021/acs.jmedchem.6b01285 28051863

[B69] Vriens J. Nilius B. Voets T. (2014). Peripheral thermosensation in mammals. *Nat. Rev. Neurosci.* 15 573–589. 10.1038/nrn3784 25053448

[B70] Wang H. Siemens J. (2015). TRP ion channels in thermosensation, thermoregulation and metabolism. *Temperature (Austin, Tex.)* 2 178–187. 10.1080/23328940.2015.1040604 27227022 PMC4843888

[B71] Wechselberger M. Wright C. L. Bishop G. A. Boulant J. A. (2006). Ionic channels and conductance-based models for hypothalamic neuronal thermosensitivity. *Am. J. Physiol. Reg. I* 291 R518–R529. 10.1152/ajpregu.00039.2006 16690776

[B72] Woo J. Jeon Y. K. Zhang Y. H. Nam J. H. Shin D. H. Kim S. J. (2019). Triple arginine residues in the proximal C-terminus of TREK K(+) channels are critical for biphasic regulation by phosphatidylinositol 4,5-bisphosphate. *Am. J. Physiol. Cell. Physiol.* 316 C312–C324. 10.1152/ajpcell.00417.2018 30576235

[B73] Woo J. Shin D. H. Kim H. J. Yoo H. Y. Zhang Y. H. Nam J. H. (2016). Inhibition of TREK-2 K(+) channels by PI(4,5)P2: an intrinsic mode of regulation by intracellular ATP via phosphatidylinositol kinase. *Pflugers Arch.* 468 1389–1402. 10.1007/s00424-016-1847-0 27283411

[B74] Yamamoto Y. Hatakeyama T. Taniguchi K. (2009). Immunohistochemical colocalization of TREK-1, TREK-2 and TRAAK with TRP channels in the trigeminal ganglion cells. *Neurosci. Lett.* 454 129–133. 10.1016/j.neulet.2009.02.069 19429069

[B75] Zhang H. Shepherd N. Creazzo T. L. (2008). Temperature-sensitive TREK currents contribute to setting the resting membrane potential in embryonic atrial myocytes. *J. Physiol. London* 586 3645–3656. 10.1113/jphysiol.2008.153395 18566002 PMC2538834

[B76] Zhao H. Sprunger L. K. Simasko S. M. (2010). Expression of transient receptor potential channels and two-pore potassium channels in subtypes of vagal afferent neurons in rat. *Am. J. Physiol. Gastrointest. Liver Physiol.* 298 G212–G221. 10.1152/ajpgi.00396.2009 19959819 PMC2822499

